# Supplementary material for: A Global Screen for Assembly State Changes of the Mitotic Proteome by SEC-SWATH-MS
Source: Cell Syst. 2020 Feb 26;10(2):133–155.e6. doi: 10.1016/j.cels.2020.01.001 (PMC7042714; doi:10.1016/j.cels.2020.01.001)

Q12981 | SEC20_HUMAN | BNIP1 NIP1 SEC20L TRG8
Monomer MW [kDa]: 26.132 Monomer expected elution fraction: 52

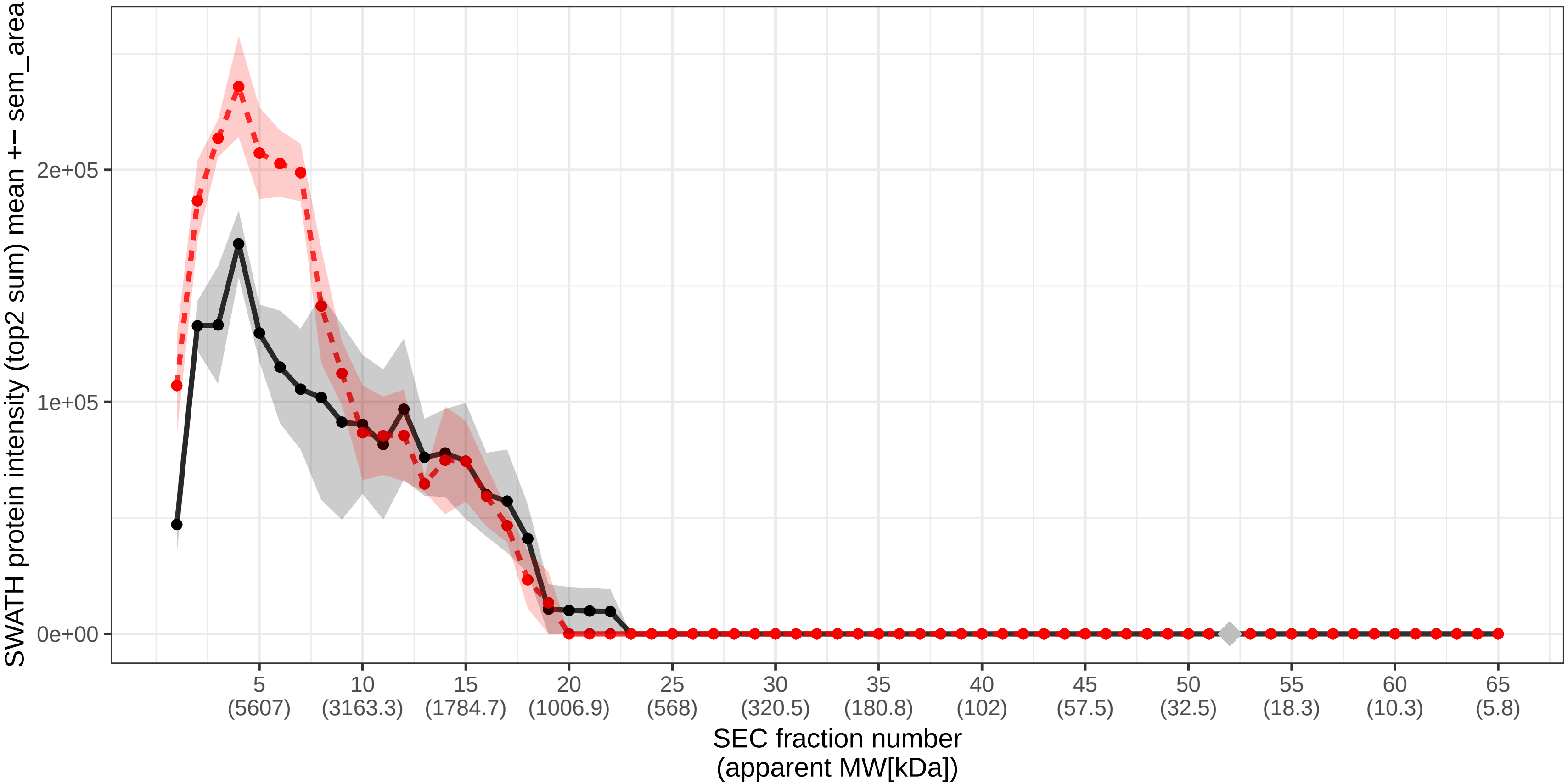

Supplement: Data S1. SEC-SWATH-MS Protein Chromatograms, Related to Figure 1 [file mmc6.zip › SECchrom_Q12981_SEC20_HUMAN_BNIP1_NIP1_SEC20L_TRG8.pdf]
